# Multifunctional Proteins Bridge Mitosis with Motility and Cancer with Inflammation and Arthritis

**DOI:** 10.1100/tsw.2010.141

**Published:** 2010-06-29

**Authors:** Jihong Jiang, Rosaely Casalegno-Garduno, Helen Chen, Anita Schmitt, Michael Schmitt, Christopher A. Maxwell

**Affiliations:** ^1^ Department of Pediatrics, Child and Family Research Institute, University of British Columbia, Vancouver, BC, Canada; ^2^ Department of Internal Medicine III, University of Rostock, Germany

**Keywords:** cancer, arthritis, mitosis, motility, RHAMM, survivin, 14-3-3, stratifin, GRP78

## Abstract

While most secreted proteins contain defined signal peptides that direct their extracellular transport through the ER-Golgi pathway, nonclassical transport of leaderless peptides/proteins was first described 20 years ago and the mechanisms responsible for unconventional export of such proteins have been thoroughly reviewed. In addition to directed nonclassical secretion, a number of leaderless secreted proteins have been classified as damage-associated molecular-pattern (DAMP) molecules, which are nuclear or cytoplasmic proteins that, under necrotic or apoptotic conditions, are released outside the cell and function as proinflammatory signals. A strong association between persistent release of DAMPs, chronic inflammation, and the hypoxic tumor microenvironment has been proposed. Thus, protein localization and function can change fundamentally from intracellular to extracellular compartments, often under conditions of inflammation, cancer, and arthritis. If we are truly to understand, model, and treat such biological states, it will be important to investigate these multifunctional proteins and their contribution to degenerative diseases. Here, we will focus our discussion on intracellular proteins, both cytoplasmic and nuclear, that play critical extracellular roles. In particular, the multifunctional nature of HMMR/RHAMM and survivin will be highlighted and compared, as these molecules are the subject of extensive biological and therapeutic investigations within hematology and oncology fields. For these and other genes/proteins, we will highlight points of structural and functional intersection during cellular division and differentiation, as well as states associated with cancer, such as tumor-initiation and epithelial-to-mesenchymal transition (EMT). Finally, we will discuss the potential targeting of these proteins for improved therapeutic outcomes within these degenerative disorders. Our goal is to highlight a number of commonalities among these multifunctional proteins for better understanding of their putative roles in tumor initiation, inflammation, arthritis, and cancer.

## CYTOSKELETAL ON THE INSIDE, CORECEPTOR ON THE OUTSIDE

### RHAMM/CD168 Binds Hyaluronan and Regulates CD44, EMT, and Tumorigenesis

The Receptor for Hyaluronan (HA) Mediated Motility (RHAMM), an acidic, coiled-coil protein designated as CD168[1], was originally identified within the conditioned media of murine fibroblasts as a component of the HA receptor complex (HARC) (reviewed in [2]). In fibroblasts, overexpression of RHAMM cDNA was transforming, while *ras*-transformed fibroblasts were reverted through expression of a dominant-negative mutant of RHAMM, which was altered in the COOH-terminal primary structure[3]. The COOH terminus of RHAMM contains a basic primary structure that mediates HA binding through an ionic interaction[4]. This domain and interaction distinguishes RHAMM from CD44, another major HA binding protein that utilizes tandem repeat loops with homology to the cartilage link protein to interact with HA (reviewed in [5]). Thus, extracellular RHAMM regulates cellular transformation and migration in an HA-dependent manner.

Given the central role that HA plays in tumor cell survival, proliferation, and invasion[5], it is perhaps unsurprising that molecular interactions between RHAMM and CD44 regulate these phenotypes. Analysis of genetically modified animals and lines revealed functional redundancy and interdependence between RHAMM and CD44 during inflammation and cell migration, respectively[6,7]. In CD44 –/– mice, collagen-induced arthritis and leukocyte infiltration is supported by RHAMM engagement of HA on the surface of splenic leukocytes[6]. In RHAMM –/– fibroblasts, migration is impaired due to reduced CD44 display at the cell surface and impaired ERK1 activation[7]; together, these effects result in impaired wound resolution[7]. Finally, secreted RHAMM binds HA and, in concert with CD44, facilitates migration of breast cancer cells[8]. In breast cancer cell lines, RHAMM-CD44 interactions are more pronounced in highly invasive lines such as MDA-MB-231[8]. Therefore, CD44-mediated migration during inflammation, wound healing, and tumorigenesis requires, or is responsive to, surface display of RHAMM. These observations are provocative given the strong associations between CD44 expression, “stemness”, and EMT/motile phenotypes within identified tumor-initiating populations[9]. Indeed, RHAMM expression augmented the tumor-initiating populations of 10T1/2 fibroblasts, while mutated RHAMM impaired tumor initiation in *ras*-transformed fibroblasts[3]; CD44 expression within these populations was not examined.

Tumor-initiating cells (TICs) are functionally classified by their amplified ability, relative to unsorted cancer cells, to generate tumors in xenograft models[10,11]. In a seminal paper, Clarke and colleagues identified tumor-initiating populations within breast cancer patients based, in part, on high expression of CD44[12]. Depending on the tumor, between 11 and 35% of Lin– cells were characterized as CD44+/CD24–[12]; more recently, CD44+/CD24– epithelial tumor cells have been related to basal-like, triple-negative breast carcinomas with poor prognosis[13]. Elevated expression of CD44 also characterizes TICs from patients with colorectal[14] and pancreatic[15] tumors. Recently, an intimate link has been drawn between CD44+ TICs and the EMT program. In fact, these two cellular phenotypes may be interchangeable as EMT generates breast cancer CD44+ TICs, while human CD44+ TICs express EMT markers[9]; activation of oncogenic *ras* may be essential to the EMT necessary for generation of CD44+ TICs[16]. It is important to note that CD44 expression may directly regulate “tumor-initiating” phenotypes rather than passively marking TICs; many of the early reports on the biology of CD44 (also known as pgp-1 and as the drug sensitivity marker mc56) demonstrated modulation of xenograft survival, cellular migration, and drug sensitivity and resistance[17,18,19]. If so, the regulation of CD44 expression, especially in the context of *ras* activation, may be an essential aspect of EMT for TICs. Given the critical role that RHAMM surface display plays in modifying CD44-induced cellular phenotypes, such as tumor initiation and motility, extracellular RHAMM may regulate CD44+ TICs and provide fundamental insight into the migration, survival, and growth of these therapeutically relevant populations.

Multiple reviews[10,20,21] have recently outlined the process of EMT during development and its potential role in cancer. However, a brief overview of the importance of EMT to tumorigenesis is warranted. In essence, metazoans consist mainly of two types of cells, epithelial and mesenchymal, which differ both in structure and function[20]. Epithelial cells are stationary, contain cell-cell junctions, and are highly polarized, while mesenchymal cells are motile, lack cell-cell junctions, and are less polarized[20]. During differentiation and, potentially, oncogenesis, these cellular states are not fixed and a cell can show plasticity, transitioning from epithelial to mesenchymal (EMT) or mesenchymal to epithelial (MET). In humans, many of the most common tumors are carcinomas, originating in epithelial cells. As these tumors often spread to distant sites within the body, it is speculated that the tumor may hijack a differentiation program that promotes a transition from epithelial-to-mesenchymal structure, including the loss of cell-cell junctions and the acquisition of motile characteristics. Alternatively, carcinomas may arise from cancer stem cell populations. This population of cells may associate with a specific differentiation state, lacking the specialized and polarized characteristics of terminal differentiated epithelia, and aligning with EMT-like characteristics[10]. It is most likely that one hypothesis for tumorigenesis will not describe all cancers, or all carcinomas; however, it is clear that metastasis is a lethal consequence of tumor progression. Cellular migration is a fundamental characteristic of metastasis and an improved understanding of the genetic, biochemical, and mechanical influences (within, supporting, and surrounding the transformed cells) that determine migration, or EMT, are important goals for oncology.

Importantly, most of the extracellular functions for RHAMM are demonstrated during inflammation, wound healing, and/or tumorigenesis. One important exception may be a recent report of RHAMM expression during the development of the nervous system in *Xenopus* embryogenesis[22]. While functional analyses were not performed, it is provocative to note that RHAMM expression correlated with the expression of CD44 and the hyaluronan synthase 1 (HAS1) in the migrating cranial neural crest[22]. Neural crest formation, along with gastrulation, is perhaps the best-studied EMT process[20]; therefore, gene expression patterns place RHAMM, CD44, and HA together during a critical EMT for normal development. Taken together, these data suggest a pivotal role for extracellular RHAMM-CD44 in determining cell fate during normal developmental processes of EMT, as well as during degenerative disease states, such as cancer, when this program may augment the survival and migration of tumor populations.

## RHAMM Assembles Microtubules and Regulates Mitosis and Differentiation

In addition to its expression during neural crest formation, RHAMM is highly expressed during proliferative processes of embryogenesis, potentially in an HA-independent manner[22]. These findings are consistent with elevated RHAMM expression during tail regeneration in *Xenopus* tadpoles, a period of intense cell proliferation[23]. Moreover, RHAMM mRNA and protein expression levels are regulated by cell-cycle progression in human cell lines[24,25], while expression of RHAMM mRNA in the adult human is restricted to proliferating tissues, like testis, tonsils, and bone marrow[26]. These expression data strongly suggest that the protein's function relates to cell division and mitosis. Indeed, RHAMM directly binds microtubules through an NH2-terminal domain and interacts with the mitotic spindle[27,28]. While the basic nature of the COOH terminus mediates an ionic interaction with HA, primary structure analysis and deletion constructs identify this region as a centrosome-targeting, basic leucine zipper motif with high homology to the microtubule motor protein KLP2[28]. In both human and *Xenopus*, RHAMM localizes to the centrosome and mitotic spindle pole through an interaction with targeting protein for XKlp2 (TPX2), while inhibition or overexpression of RHAMM disrupts mitotic spindle assembly[24,29]. Consistently, RHAMM –/– fibroblasts contain abnormal mitoses in culture[7]. Finally, recent screens[30,31] have identified key roles for RHAMM in spindle disassembly and mitotic exit. Together, these data support a critical role for RHAMM in the formation of the mitotic apparatus as a microtubule-associated protein.

Many of the "hallmarks of cancer", including self-sufficiency in growth signals, insensitivity to antiproliferative signals, and limitless replicative potential, suggest that cancer is a disease of altered cell proliferation[32]. For this reason, an understanding of the mechanisms responsible for mitotic spindle assembly and exit are essential to the study and targeting of cancers. Moreover, microtubule dynamics play an integral role in cellular differentiation and migration. That is, the nucleation of microtubules at cell-cell junctions is essential to epithelial structure[33], while the positioning of the microtubule organizing center, the centrosome, is a determining factor in mesenchymal migration[34]. Thus, structural cues from microtubule organization likely play prominent roles in cancer progression, during both proliferation and migration.

Cell division is one of the most-studied cellular processes (175,000 PubMed citations) and the protein regulators of microtubule organization during mitosis have been well described. In general, the formation of the mitotic spindle requires two microtubule-centered processes, microtubule assembly and microtubule capture. Microtubule assembly occurs at centrosomes/spindle poles and proximal to chromosomes, while microtubule capture occurs at kinetochores. Broadly speaking, the aurora kinases regulate these two processes with aurora kinase A (AURKA) promoting microtubule assembly and aurora kinase B (AURKB) promoting microtubule capture[35,36]. RHAMM and TPX2 are nonmotor spindle assembly proteins that regulate microtubule assembly and cross-linking during spindle formation (reviewed in [2,37]). TPX2 is a potent activator of AURKA[38], and RHAMM regulates TPX2 localization[24,29] and influences microtubule assembly. In turn, RHAMM abundance is determined by the activity of the heterodimeric E3 ubiquitin ligase, Breast Cancer, early onset 1 (BRCA1)–BRCA1-associated RING domain 1 (BARD1)[39,40]. Thus, RHAMM abundance regulates TPX2 and is regulated by BRCA1-BARD1, as well as the anaphase-promoting complex[31], to influence microtubule nucleation through AURKA. AURKA-BRCA1-RHAMM-TPX2 interactions, which regulate microtubule organization during mitosis, may be conserved during differentiation and migration..

Given the correlation between RHAMM expression, proliferation, and EMT during *Xenopus* development, RHAMM may regulate microtubule organization during division and differentiation (e.g., EMT or MET). Importantly, cytoskeletal changes regulate both EMT and MET. Apico-basal polarization of epithelial cells (i.e., MET) involves a dramatic reorganization of the microtubule cytoskeleton[41], while cytoskeletal changes are crucial for these cells to leave the epithelium and begin migrating individually during EMT[20]. In particular, the dissolution of adheren junctions is an essential event to disrupt epithelial cell polarity[20]. Microtubule nucleation is perhaps an underappreciated role for adheren junctions in epithelia[33,42] and dissolution of these sites may promote centrosome nucleation of microtubules, which highlights a fundamental similarity between EMT and mitotic spindle assembly.

AURKA activation is vital for the reorganization of the microtubule cytoskeleton in polarized, nonmitotic epithelia[43]. AURKA promotes interphase microtubule nucleation through the phosphorylation of BRCA1, down-regulating BRCA1-BARD1 ubiquitination of centrosome proteins, and increasing the nucleation potential of the centrosome[44,45]. In human carriers of BRCA1 mutations, luminal differentiation of mammary progenitors is altered[46], supporting the hypothesis that BRCA1 determines stem/progenitor cell fate[47]. Indeed, site-directed mutagenesis of the AURKA phosphorylation site in BRCA1 is sufficient to alter the differentiation potential of embryonic stem cells[48]; together, these data highlight a fundamental role for AURKA and BRCA1 during differentiation. Given that RHAMM regulates mitotic organization of microtubules through AURKA-BRCA1[39,40], intracellular regulation of cytoskeletal elements by AURKA, BRCA1, and RHAMM may play vital roles in cellular fates, such as luminal differentiation and EMT. Consistently, disruption of AURKA[49], BRCA1[50], or RHAMM[51] modifies neurite extension, an alternate differentiation program dependent on microtubule nucleation.

Thus, RHAMM is an example of an intracellular cytoskeletal protein that regulates differentiation and division, and, upon release or secretion, alters cellular responses to extracellular cues, such as HA. In this way, RHAMM secretion may be an active process of tumor cells that promotes “tumor-initiating” properties, such as EMT and migration, and/or RHAMM release may be an indicator of degenerative disease, such as cancer, that modulates immune responses.

## Secreted, Cytoskeletal Proteins in Degenerative Disease

RHAMM is not alone in its function as an intracellular, cytoskeletal protein that, upon release, regulates extracellular processes (Fig. 1). Glucose-regulated protein (GRP)-78 and GRP-75 are molecular chaperone proteins that interact with RHAMM at microtubules[52] and, like RHAMM, are molecular components of the *Xenopus* Meiotic Microtubule-Associated Interactome[53]. GRP-78, aka "immunoglobulin heavy chain-binding protein" or Bip, was identified from cells starved of glucose and is a heat-shock protein 70 family member that relocalizes to the cell surface in response to ER stress[54]. Similar to RHAMM, the roles for extracellular GRP-78 are expanding to include the regulation of cancer cell susceptibility to drugs[55], and the regulation of ERK and PI3K signaling pathways through a coreceptor[56]. Moreover, a strong association has been drawn between GRP-78 secretion, adipocyte and osteoblast differentiation[57]. Finally, the anti-inflammatory and immune-modulatory properties of extracellular GRP-75 have been reviewed[58] making this molecule, like RHAMM, an emerging therapeutic target for the treatment of arthritis[59].

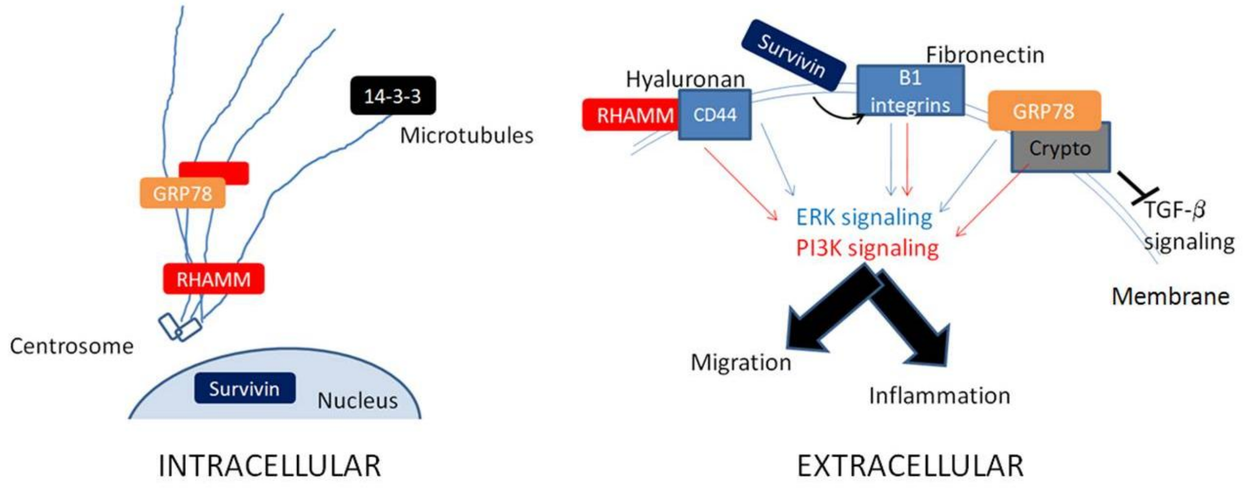

**FIGURE 1.** Cytoskeleton on the inside, coreceptor on the outside. Many cytoskeletal and nuclear proteins function as coreceptors within degenerative disease, such as cancer and arthritis. Frequently, these secreted proteins influence signaling through ERK, PI3K, and NF-kB (not shown) with downstream consequences on cell migration and inflammation.

Moreover, the 14-3-3 proteins are acidic, coiled-coil, scaffold proteins that interact with a variety of partners and influence signaling pathways, cell cycle checkpoints, and DNA damage responses (reviewed in [60,61]). 14-3-3 proteins localize to centrosomes and mitotic microtubules[62], and14-3-3 sigma, aka stratifin or interferon regulatory factor 6 (Irf6), plays a vital role in the prevention of mitotic catastrophe after DNA damage[63,64]. Stratifin, an antifibrogenic factor found in conditioned media from keratinocytes[64,65,66,67], is a key determinant of the proliferation-differentiation switch in keratinocytes[68]. Therefore, RHAMM, GRP-78/Bip, and stratifin represent cytoskeletal proteins with vital roles in division and differentiation; under conditions wherein the balance of differentiation and division is disrupted (i.e., cancer), these proteins are secreted or released into the extracellular compartments and regulate key aspects of cellular migration, tumor initiation, inflammation, and fibrosis. Consequently, these multifunctional proteins may be novel targets within degenerative disease (Table 1).

**TABLE 1**
**Multifunctional Proteins and Degenerative Disease**

| ***Gene*/Protein** | **Intracellular Function** | **Extracellular Function** | **Role in Degenerative Diseases** |
|---|---|---|---|
| *Birc5*/Survivin | Cytoplasm/mitochondria: inhibits apoptosis via mitotic microtubules[107,108] and caspase-3, -7, and -9 activity[109,110]<br>Mitosis: controls cell division as a subunit of the chromosomal passenger complex[108,111] | Survivin signals extracellularly through p38 MAPK, leading to the expression of beta2-integrins, significance for the pathogenesis of inflammation in arthritis[85]; cancer cells absorb cell-permeable survivin to enhance growth and metastasis[84] | Cancer: ubiquitous elevation in most solid and blood tumors[112]<br>Arthritis: high levels of extracellular survivin are associated with chronic erosive rheumatoid arthritis[90] |
| *HMMR*/RHAMM | Microtubule-associated protein that maintains mitotic spindle pole integrity through dyenin[28] | RHAMM-CD44 complex regulates cellular transformation and migration in an HA-dependent manner[8] | Cancer: elevation in most solid and blood tumors[2]<br>Arthritis: aggressive inflammatory response in rheumatoid arthritis[6] |
| *HSPA5*/GRP78, Bip | ATP-dependent chaperone assists folding of newly synthesized polypeptides, the assembly of multiprotein complexes, the transport of proteins across cellular membranes[113], protects from ER stress[114]; blocks apoptotic pathway at different levels and rescues cells from late phase of apoptosis[115,116] | Extracellular or membrane-bound GRP-78 can elicit an immune response either by the adaptive or innate immune system[115], and acts as carriers for antigenic peptides derived from tumor cells, which is internalized via HSP receptors[117] | Cancer: elevation in most solid and blood tumors[115]<br>Arthritis: up-regulation in rheumatoid arthritis[118] |
| *SFN*/Stratifin, 14-3-3 sigma | Induces conformational changes in binding partners that alter enzymatic activities, localization, stability, and phosphorylation state[119]<br>Ex. induces G2/M arrest by binding and sequestering Cdc2-cyclinB complexes[120] | Key determinant in the switch between proliferation and differentiation in keratinocytes[65] | Cancer: levels are reduced/absent due to promoter methylation in epithelia cancers, participate in G2/M checkpoint control[120]<br>Neurological disorders: facilitate Tau phosphorylation[121] (Alzheimer's); association with alpha-synuclein inhibit its antiapoptotic function[122](Parkinson's) |

## SURVIVIN: DIVISION, DEATH, AND ARTHRITIS

In addition to cytoskeletal proteins, release or secretion of nuclear proteins has also been implicated in inflammation, cancer, and arthritis[69]. Survivin, a member of the inhibitor of apoptosis (IAP) gene family, is a small protein with a COOH-terminal, coiled-coil structure that localizes to multiple intracellular compartments, including nuclei, mitochondria, and mitotic centrosomes and microtubules (reviewed in [70]). In addition to its cell-protective roles, survivin regulates microtubule dynamics and is an essential mitotic gene[71]. Like RHAMM[24,28,29], knockdown of survivin induces mitotic defects[72] with both molecules influencing ran-mediated spindle assembly through TPX2[39,73]. Further to microtubule assembly, survivin plays a key role in microtubule capture at kinetochores. Survivin regulates the localization of the inner centromere protein (INCENP)[72,74,75], a key activator of AURKB, while RHAMM regulates the localization of TPX2, a key activator of AURKA. Just as RHAMM ubiquitination by BRCA1-BARD1 regulates spindle assembly[39], ubiquitination/ deubiquitination of survivin by hFAM regulates chromosome alignment and segregation[76]. Therefore, these microtubule regulators are important determinants of correct cell division.

The microtubule-associated effects(s), if any, of survivin during cellular differentiation are unknown. However, a number of interesting parallels between survivin and RHAMM suggest a role for survivin in epithelial differentiation, EMT, and neurite outgrowth. In the human, survivin expression, like RHAMM, is restricted to tissues with proliferative potential, like the thymus and testis[77]. In the mouse embryo, survivin expression is nearly ubiquitous at embryonic day (E) 11.5, but restricted at E15 to -21 to the distal bronchiolar epithelium of the lung and neural crest–derived cells[78]. These expression data are suggestive of a role in proliferation, death, and EMT. Consistently, endothelial-specific loss of survivin results in embryonic lethality due to a lack of normal EMT and neural tube closure defects[79]. Thus, RHAMM and survivin function may converge during embryonic EMT. In the adult rodent, both molecules may contribute to neurite outgrowth. In PC12 cells, neurite outgrowth is suppressed by HA signaling through RHAMM[80] and by overexpression of NAIP-2, a structurally related protein to survivin in rodents[81]. It is most likely that survivin regulates cellular differentiation and death through intracellular functions; however, recent work in models of cancer and inflammation suggest additional, extracellular roles for survivin, similar to RHAMM.

We speculate that an elevated mitotic rate within cancer may lead to surface display of RHAMM and survivin. If so, expression of these genes/proteins may be correlated across cancer sites. Indeed, in the Bittner multicancer dataset, including analysis of over 19,000 genes within 213 cancers (1911 samples), the expression of *HMMR*/RHAMM was most strongly correlated with BIRC5/survivin, along with 14 other genes, including TPX2 (Fig. 2)[26]. These results were confirmed in the Janoueix-Lerosey Brain dataset, over 19,000 genes within 64 samples (Fig. 2)[26,82]. Functional similarities between coexpressed genes revealed a strong bias towards mitotic genes/proteins with associations to microtubules and aurora kinase activity (Fig. 2). However, many coexpressed genes have also been implicated in tumor outcome and metastasis. Survivin, for example, regulates the migratory capacity of invasive breast cancer cell lines and its overexpression is sufficient to increase metastatic potential through up-regulation of fibronectin[83]. It is yet unclear whether extracellular survivin, like RHAMM, regulates tumor cell migration and survival or inflammation to the tumor site.

Like RHAMM and stratifin, survivin has been identified in the conditioned media of tumor cell lines[84]. Extracellular survivin protected cancer cells from apoptosis and genotoxic stress, and promoted migratory behavior[84]. Indeed, survivin may promote cell signaling cascades as part of its protective and migratory effects. In leukocytes, extracellular survivin induces integrin expression through p38 MAPK and PI3K signaling[85]; these modifications are strikingly similar to the effects of extracellular RHAMM on beta-integrins in thymocytes[86] and the effect of GRP-78/Bip on Cripto signaling in cancer cells[56]. Another striking parallel between these intracellular proteins is their association and/or detection with inflammatory disorders, such as arthritis.

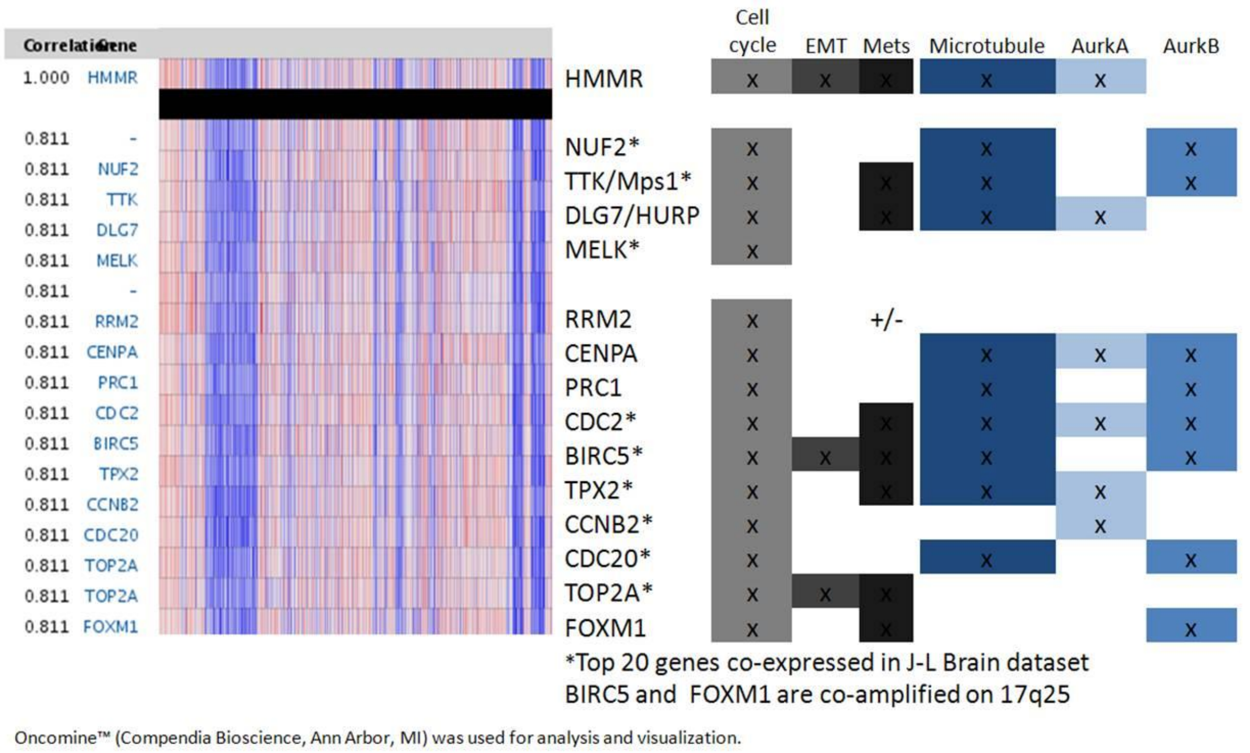

**FIGURE 2.** Coexpression of mitotic/metastasis genes in cancer. Expression of *HMMR*/RHAMM correlates to expression of other mitotic genes across multiple tumor sites analyzed in the Bittner[26] and Janoueix-Lerosey Brain[82] datasets. Coexpressed genes/proteins associate with microtubules and interact with aurora kinases. Additionally, expression of these genes/proteins associate with cellular migration, EMT, and adverse tumor outcomes, such as metastasis (Mets). Oncomine™ (Compendia Bioscience, Ann Arbor, MI) was used for analysis and visualization.

## Intracellular Proteins, Inflammation, and Arthritis: Autoantibodies Point the Way

Thus far, we have reviewed evidence for secretion of intracellular regulators of mitosis and differentiation in the determination of cellular migration, tumorigenesis, and inflammation. The extracellular secretion of these molecules may be restricted to degenerative states, such as cancer and arthritis, and key developmental processes during embryogenesis. Consistently, extracellular RHAMM, GRP-78/Bip, 14-3-3, and survivin are associated with inflammatory disorders, such as arthritis. If so, the secretion of these molecules may illicit immune responses that not only demarcate degenerative disease, but also provide new therapeutic targets.

The presence and functions of these multifunctional proteins have been examined both in diseased human tissues as well as in animal models of disease. For example, RHAMM, as detected by immunohistochemistry and quantitative immunoblot, is significantly elevated in knee synovial tissue of patients with advanced osteoarthritis (OA) compared to those without[87]. These results are consistent with animal model studies demonstrating an isoform-specific role for RHAMM in collagen-induced arthritis (CIA)[6]. Examination of synovial fluid (SF) of patients with inflamed joints revealed the presence of stratifin, along with 14-3-3 eta, which correlated to the levels of defined biomarkers for rheumatoid arthritis (RA)[88]. Extracellular survivin has also been detected in the SF of patients with RA; survivin levels were significantly higher in RA than OA and were higher still in patients with erosive RA compared to nonerosive RA[89]. It is postulated that extracellular survivin may be produced by rheumatoid fibroblast-like synoviocytes and induce apoptotic resistance within these cells, provoking a positive feedback on their proliferation[89]. Not only has extracellular survivin been detected in the SF of patients with RA, and associated with destructive disease, but antibodies to survivin relate to nonerosive

RA[90]. Therefore, many multifunctional extracellular molecules impact inflammation and arthritis. Some of these molecules, like Bip, are anti-inflammatory, while others, like survivin and RHAMM, play proinflammatory roles. The modulation of these dynamics may dramatically alter disease. Finally, the presence of these molecules can be detected indirectly by the examination of humoral responses, and may present therapeutic options in arthritis and cancer.

In addition to RHAMM, autoantibodies against survivin (reviewed by [91]) and 14-3-3 theta[92] have been detected in various types of cancer. Moreover, studies have shown an association of humoral immune response with tumor progression and prognosis (reviewed by [91]). Although autoantibodies have appealing features as biomarkers (such as high specificity, simple detection techniques, and persistent presence in serum[93]), they are limited by moderate sensitivity. Only 10–20% of patients with survivin overexpression will develop survivin antibodies (reviewed by [91]). An effective way to overcome this disadvantage lies in the simultaneous assessment of a panel of autoantibodies for each type of tumor[94]. Like survivin, RHAMM has been identified by SEREX as a tumor-associated antigen in a panel of tumors[95,96]. Given the strong correlation between survivin/RHAMM in the states of inflammation, arthritis, and cancer, they could be good candidates for the panel of biomarkers. The utility of these humoral responses as markers for tissue damage and/or degenerative disease must be examined.

Finally, emerging cell-based strategies to target these molecules in cancer have shown promising preclinical and clinical results. For example, peptide vaccination with a RHAMM-derived, highly immunogenic peptide, termed R3, has proven safe and effective at generating CD8+ cytotoxic cellular responses and antitumor activity in patients with acute myeloid leukemia, myelodysplastic syndrome, multiple myeloma, and, more recently, chronic lymphocytic leukemia[97,98]. An alternate strategy of vaccination with dendritic cells expressing exogenous RHAMM mRNA has proven effective against murine models of glioma[99]. Similarly, a survivin peptide-based vaccination proved safe in patients with advanced or recurrent colorectal and urothelial cancer[100,101], while survivin-expressing dendritic cells demonstrate antitumor responses in experimental models[102,103]. Provocatively, vaccination of a murine model for neuroblastoma with survivin minigene DNA demonstrated significant antitumor activity[104]. These cell-based therapies may show tumor-specific activities; with this in mind, neuroblastoma may be sensitive to RHAMM-based immunotherapies given the established roles of AURKA[105] and BARD1[106], two components of the AURKA-BRCA1/BARD1-RHAMM-TPX2 centrosome module, within this refractory disease.

## ACKNOWLEDGMENTS

This work is supported by a Child & Family Research Institute Establishment Award (CAM), the Michael Cuccione Foundation (CAM), and continuous support by the German José Carreras Leukemia Foundation, in particular through the generous grants R 09/14 and R10/03 (MS).
